# Selective Decontamination of the Digestive Tract in Pancreatic Head Resections—A Propensity Score-Matched Analysis

**DOI:** 10.3390/jcm12010250

**Published:** 2022-12-29

**Authors:** Olga Radulova-Mauersberger, Florian Oehme, Alexandra Doerell, Laura Frohneberg, Sebastian Hempel, Jürgen Weitz, Thilo Welsch, Marius Distler, Christian Teske

**Affiliations:** 1 Department of Visceral-, Thoracic and Vascular Surgery, University Hospital Carl Gustav Carus, Technische Universität Dresden, 01307 Dresden, Germany; 2 National Center for Tumor Diseases (NCT/UCC), Faculty of Medicine and University Hospital Carl Gustav Carus, Technische Universität Dresden; German Cancer Research Center (DKFZ), Helmholtz-Zentrum Dresden-Rossendorf (HZDR), 01307 Dresden, Germany; 3 Department of General, Visceral and Thoracic Surgery, St. Elisabethen-Klinikum Ravensburg, Academic Teaching Hospital of the University of Ulm, Elisabethenstr. 15, 88212 Ravensburg, Germany

**Keywords:** selective bowel decontamination, pancreatic surgery, HPB surgery, POPF, bile duct

## Abstract

(1) Background: The postoperative morbidity rate after pancreatic head resection remains high, partly due to infectious complications. The primary aim of this study was to analyze the influence of selective decontamination of the digestive tract (SDD) on the postoperative infection rate after pancreatic surgery. (2) Methods: From January 2019, the standard of care for patients undergoing pancreatic head resections at the Department for Visceral, Thoracic, and Vascular Surgery, University Hospital Dresden was the preoperative oral administration of SDD. The influence of SDD was evaluated for patients operated on between January 2019 and June 2020 in comparison to a propensity score-matched cohort, extracted from an existing database including all pancreatic resections from 2012 to 2018. The primary endpoint of the study was the shift of the bacterial load on the intraoperative bile swab test. The secondary endpoint was the association of SDD with postoperative complications. (3) Results: In total, 200 patients either with SDD (*n* = 100; 50%) or without SDD (non-SDD, *n* = 100; 50%) were analyzed. In the patient group without a preoperative biliary stent, 44% (*n* = 11) of the non-SDD group displayed positive bacterial results, whereas that was the case for only 21.7% (*n* = 10) in the SDD group (*p* = 0.05). Particularly, Enterobacter species (spp.) were reduced from 41.2% (*n* = 14) (non-SDD group) to 23.5% (*n* = 12) (SDD group) (*p* = 0.08), and Citrobacter spp. were reduced by 13.7% (*p* = 0.09) from the non-SDD to the SDD cohort. In patients with a preoperative biliary stent, the Gram-negative Enterobacter spp. were significantly reduced from 52.2% (*n* = 12) in the non-SDD group to 26.8% (*n* = 11) in the SDD group (*p* = 0.04). Similarly, Citrobacter spp. decreased by 20.6% from 30.4% (*n* = 7) to 9.8% (*n* = 4) in the non-SDD compared to the SDD group (*p* = 0.04). In general, deep fluid collection and abscesses occurred more frequently in the non-SDD group (36%; *n* = 36 vs. 27%; *n* = 27; *p* = 0.17). (4) Conclusions: Adoption of SDD before pancreatic head surgery may reduce the bacterial load in bile fluid. SDD administration does not significantly affect the postoperative infectious complication rate after pancreatic head resections.

## 1. Introduction

The importance of the gut microbiome in upper gastrointestinal (GI) surgery is still a poorly explored topic in visceral surgery. Several studies indicate that surgery may cause a strong shift in the gut microbiome, but the impact of these changes is not yet well-understood [1].

There is strong evidence for the benefit of selective decontamination of the digestive tract (SDD) in colorectal surgery in preventing septic complications, such as anastomotic insufficiency, pneumonia, and surgical site infections (SSI), with consequently reduced morbidity [2,3,4,5].

Some reports demonstrated the advantage of SDD being even higher when combined with mechanical bowel preparation (MBP) for the reduction of postoperative morbidity and severe complications in colorectal surgery [5]. However, evidence for the upper GI tract is very scarce, and the effect of SDD in hepato-pancreato-biliary (HPB) surgery has barely been investigated to date.

Despite the constant improvements in operative techniques and the considerable reduction in morbidity after pancreatic resections, postoperative infectious complications (e.g., deep organ abscesses and anastomotic leakages) and prolonged hospital stays are still unsolved problems [6]. The risk of septic complications in pancreatic surgery is even enhanced by the frequent bacterial contamination of the bile fluid, due to the preoperative biliary stent (PBS) implantation for cholestasis [7].

As the effect of SDD in pancreatic surgery is not well-examined yet, the purpose of our study was to extend the findings of previous studies for the benefit of SDD in colorectal surgery and to analyze its impact on HPB surgery. We aimed to investigate the influence of preoperative SDD administration on the bacterial spectrum of the bile tract. In addition, we explored the patients’ postoperative complications and morbidity.

## 2. Materials and Methods

### 2.1. Basic Study Characteristics

This article was written in accordance with the STROBE statement [8]. The experimental protocol was approved by the local ethics committee of the Technische Universität (TU) Dresden (BO-EK-60022022). This study was a propensity score-matched analysis. The trial cohort comprised all pancreatic head resections between January 2019 and June 2020 at the Department of Visceral, Thoracic, and Vascular Surgery, University Hospital Carl Gustav Carus, Technische Universität Dresden, Germany. During this period, continuous SDD administration was carried out for all patients prior to pancreatic surgery (SDD group). The corresponding, propensity score-matched patient cohort was defined from an existing database. This database included all pancreatic head resections from January 2012 to August 2018 (non-SDD group).

Patients were eligible for inclusion if they had undergone a pancreatic head resection (either duodenum-preserving procedure [DPPHR], pylorus-preserving pancreatic head resection [PPPD], or classic pancreaticoduodenectomy [cPD]) for pancreatic and periampullary carcinoma, cystic pancreatic lesions, chronic pancreatitis, or benign tumors.

Basic patient characteristics included median age, gender, body mass index (BMI), and physical status, according to the American Society of Anesthesiologists (ASA) [9]. The patient cohorts were matched at random during the analyzed period, using the basic patient characteristics. All medical records were extracted from electronic patient files by experienced surgeons.

### 2.2. Preoperative Selective Decontamination of the Digestive Tract

Preoperative SDD was first initiated and set as the standard of care in our department in January 2019. Patients were informed about the off-label use of SDD prior to surgery and provided written informed consent. The antibiotic bowel preparation consisted of an antibiotic/antimycotic rinsing solution with four different antibiotics: Amphotericin B, Colistin, Tobramycin, and Vancomycin. This antibiotic solution consisted of 2.5 g Amphotericin B, 1 g Colistin, 800 mg Tobramycin, 1 g Vancomycin, and 20 mL NaCl (0.9%). Two doses of this solution were administered to the patient the day before and one dose in the morning of the index operation. The administration was stopped and not continued in the postoperative course.

### 2.3. Additional Antibiotic Application and Intraoperative Bile Smear Test

According to our clinic’s standard protocol, patients planned for pancreatic resection received intravenous antibiotic prophylaxis with Piperacillin/Tazobactam 4 g/0.5 g 30–60 min before skin incision. In case of a Penicillin allergy, Clindamycin and Metronidazol were administered. Until 2019, the standard antibiotic prophylaxis was Cefuroxim 1.5 g and Metronidazol 0.5 g, and it was switched to the above-mentioned combination during the same year.

Intraoperatively, a biliary tract swab smear test was routinely preserved for microbiological examination after the transection of the choledochal or hepatic duct, respectively.

### 2.4. Postoperative Management

Postoperatively, patients with infectious complications received empiric antibiotics or a treatment according to the resistogram.

In accordance with the standard operating procedure (SOP) of our institution, patients were mobilized postoperatively on the day of surgery or, at the latest, on postoperative day 1. In a regular course, the progressing diet continued up to postoperative days 3–4. Drains were removed if secretion was normal (amylase < 10 µmol/L and daily volume < 800 mL of serous fluid) on day 3 after surgery.

### 2.5. Primary Endpoint

The primary endpoint for this study was the shift in the bacterial spectrum in the bile cultures of intraoperative smear tests. The bacterial spectrum was analyzed for all patients and separately examined for patients with a preoperative bile stent. Bacterial load was defined as “none”, “occasional”, “numerous”, or “massive” according to the microbiological report.

The smears from the bile duct were analyzed semi-quantitatively at our institution as smears on microbiological Petri plates. Each plate was divided into three sectors, and a small volume of sample was spread. If there were microbial colonies only in the first sector, then we judged it as “occasional”, if microbial colonies grew in the first and second sector, it was “numerous”, and if they grew also in the third sector, they were judged as “massive”.

### 2.6. Secondary Endpoint

The secondary endpoint was the overall morbidity defined according to the Clavien–Dindo classification (CDC) and classified as major complications (CDC > 2) [10]. These included delayed gastric emptying (DGE), postoperative POPF (POPF), and post-pancreatectomy hemorrhage (PPH), which were set and graded according to the consensus definitions of the International Study Group of Pancreatic Surgery [11,12,13]. Furthermore, data on the length of hospital stay (LOS) and duration of intensive care unit (ICU) stay were extracted from the patient files.

### 2.7. Statistical Analysis

Data were analyzed using SPSS version 26 software (IBM Corp., Armonk, NY, USA). The normality of continuous data was assessed using the Kolmogorov–Smirnov test and by inspecting the frequency distributions. Variance homogeneity was tested using Levene’s test.

The first step was to match the two patient cohorts. We performed a propensity score-matched analysis between the SDD and non-SDD groups using the nearest neighbor method to a 1:1 ratio. Propensity score deviation width was set to a threshold of < 0.2. Variables used for matching were age, sex, ASA, BMI, smoking, and diabetes. To detect residual imbalances after matching, we employed a standardized mean deviation analysis.

A competitive analysis for categorical variables between the cohorts was performed to compare baseline characteristics between the SDD and non-SDD groups using the chi-square test or Fisher’s exact test. For continuous variables, Student’s t-test, or the Mann–Whitney U test was performed where appropriate, and the results were represented as median and interquartile range (IQR).

A *p* value of <0.05 was considered the threshold of statistical significance for the whole study. During the analysis, missing data were treated as missing completely at random for the appropriate analysis. Thus, a complete case analysis was performed, and some patients were excluded.

## 3. Results

### 3.1. Description of the Study Cohort

In total, 200 patients were analyzed in this single-center propensity score-matched analysis, consisting of 100 patients who received SDD and 100 patients without decontamination. After matching, the SDD and non-SDD cohorts were comparable and differed in none of the basic characteristics significantly.

Both cohorts consisted of 61 male and 39 female patients (Table 1). The median age was 65.3 (IQR: 56.9–73.6; non-SDD) and 66 (IQR 56.3–76; SDD) years, respectively. There were more smokers within the SDD group (*p* = 0.44).

Considering the total study cohort, 70 patients (35%) were diagnosed with diabetes prior to surgery, and 46 patients (23%) were insulin-dependent. Due to cholestasis, 38 patients from the non-SDD group and 43 patients from the SDD group received a biliary drainage prior to surgery (*p* = 0.47). Pancreatic head resection was performed for malignancy in 74% of the SDD and in 68% of the non-SDD group (*p* = 0.08). However, 19% of the complete study cohort had undergone surgery for pancreatitis (17% non-SDD, 21% SDD) (Table 1).

### 3.2. Primary Endpoint: Intraoperative Bile Duct Smear Test/Bacterial Differentiation

According to the current standard operating procedure in our department, a bile duct smear test for bacterial differentiation is routinely performed. However, in only 50% of the matched patient cohort, a smear test result was recorded in the retrospective analysis (Table 2). A positive smear test for bacterial contamination of the bile was more likely to be found in the non-SDD group (68%) compared to the SDD group (58.6%; *p* = 0.28). Moreover, the median total number of different bacterial species in the bile duct was reduced from 2 (non-SDD) to 1 (SDD, *p* = 0.04). Considering only patients without preoperative bile duct stenting for cholestasis or stenosis, 44% of the non-SDD patients displayed positive bacterial results, whereas that was the case for only 21.7% in the SDD group (*p* = 0.05).

### 3.3. Bacterial Load and Bacterial Differentiation

A massive bacterial load in the bile duct smear test was seen in 52% of the non-SDD group and only 39.1% of the SDD group. However, when considering all groups of bacterial loads, there was no significant difference revealed (*p* = 0.37).

**Figure 1:**
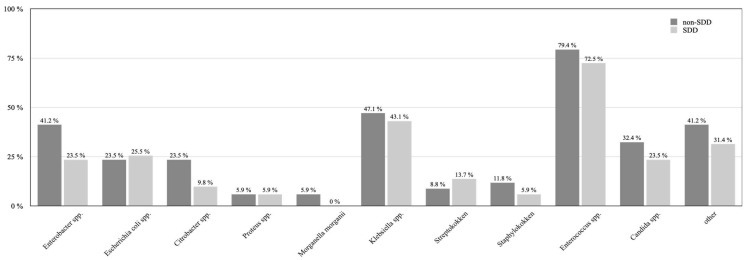
Bacterial differentiation of the intraoperative bile duct smear test in SDD and non-SDD patients is displayed.

Regarding the bacterial differentiation of the positive bile duct smear tests, there were several obvious trends towards less bacterial contamination (Figure 1). Enterobacter species (spp.) were reduced from 41.2% in the non-SDD group compared to 23.5% in patients receiving SDD (*p* = 0.08). Similarly, Citrobacter spp. were reduced from 23.5% to 9.8% in patients receiving SDD (*p* = 0.09). A similar result was revealed in Citrobacter spp., which were reduced from 23.5% to 9.8% in patients who received SDD (*p* = 0.09). Even in mycotic contamination of the bile duct with Candida spp., there was a decrease from 32.4% (non-SDD) to 23.5% (SDD; *p* = 0.37).

None of these results reached the predefined level of significance.

**Figure 2:**
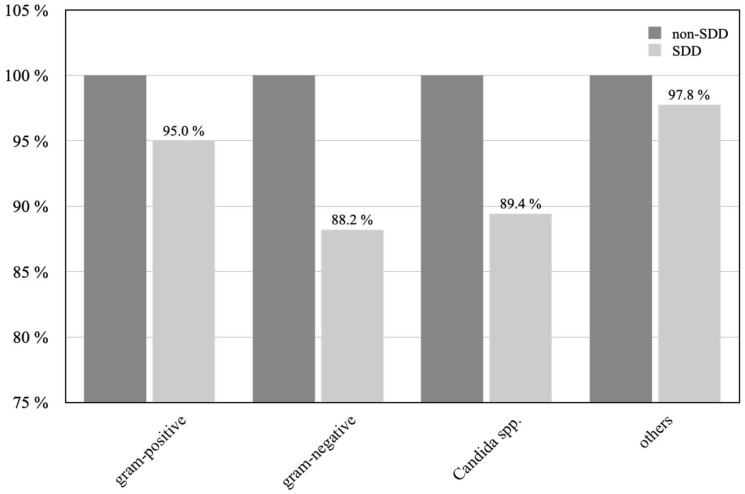
Bacterial shift between SDD and non-SDD patients is displayed according to their Gram-staining behavior. Non-SDD patients are set as 100%.

In general, Gram-positive bacteria were reduced by 5.0% (Figure 2). After SDD administration, Gram-negative bacteria and Candida spp. were decreased by 11.8% and 10.6%, respectively. Similarly, other not-specified bacterial contamination was shifted downwards by 2.2% after the introduction of SDD (Table 2).

### 3.4. Intraoperative Bile Duct Smear Test of Stent Patients

**Figure 3:**
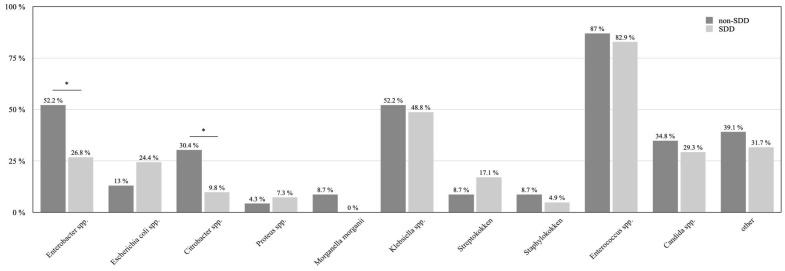
Bacterial differentiation from the intraoperative smear test of bile duct in SDD and non-SDD patients with preoperative bile duct stenting is displayed. “*”shows the significant reduction of Enterobacter spp. (*p* = 0.04) and Citrobacter spp. (*p* = 0.04) in the SDD group.

Using the data of the general bile duct smear test, a subgroup analysis was performed for patients with a preoperative bile duct stent for stenosis or cholestasis. In total, 38 patients from the non-SDD group and 43 patients from the SDD group (*p* = 0.47) received preoperative stenting. A total of 60.5% of the non-SDD patients had a positive smear test for bile contamination, whereas 95.3% of the patients in the SDD cohort had a positive test (*p* = 0.07). Due to the small number of patients, a more detailed analysis considering duration prior to surgery and stent material was not performed. Regarding the bacterial differentiation of these positive smear tests, Gram-negative Enterobacter spp. were significantly reduced from 52.2% in the non-SDD group to 26.8% in the SDD group (*p* = 0.04; Figure 3). Similarly, Citrobacter spp. were decreased by 20.6% from 30.4% to 9.8% in the non-SDD to the SDD group (*p*= 0.04). All other bacterial results are displayed in Figure 3.

### 3.5. Outcome Analysis

In the short-term outcome analysis, general postoperative complications between the SDD and non-SDD groups did not differ significantly, with 49% and 46% (*p* = 0.67), respectively (Table 3). However, a detailed analysis of the LOS and ICU stay revealed a significant shift. SDD patients were discharged after 14 days (IQR 10–22.8) compared to 19 days (IQR 14–30) for the non-SDD group (*p* < 0.001). Furthermore, ICU stay was reduced from 4 days (non-SDD, IQR 2–6.5) to 2 days (SDD, IQR 1–3; *p* < 0.001), and the readmission rate to the ICU was unaltered (16 vs. 17 days, *p* = 0.85). Interestingly, postoperative morbidity (CDC > 2) was not seen to change, with 49% of the non-SDD and 46% of the SDD group (*p* = 0.67). The in-hospital mortality rate was 8% in both cohorts. However, both 30-day and 90-day mortality rates were slightly increased in patients who received SDD (16% and 22%) compared to the propensity score-matched cohort (8% and 14%; *p* = 0.08; *p* = 0.14), but they did not reach a significant level. Surgical site infection (*p* = 0.5) and the procedure-specific complications, such as POPF (*p* = 0.31), PPH (*p* = 0.34), and DGE (*p* = 0.74), were not significantly different in either group. However, a mild shift towards higher numbers in SDD patients was seen.

Interestingly, the infectious complication of deep organ abscesses and fluid collection was slightly reduced in patients who received SDD compared to the non-SDD group, with 27% and 36% (*p* = 0.17), respectively.

## 4. Discussion

The present study evaluates the initial results after establishing a preoperative SDD standard protocol for patients with pancreatic resections in our institution. The perioperative administration of SDD has been broadly adopted for colorectal surgical procedures over recent years [2,3,4,5]. Benefits in reducing SSIs and the incidence of a postoperative anastomotic insufficiency (AI) were shown in a small number of randomized controlled trials [14,15,16]. In particular, the incidence of AI was reduced from 13.1% to 5.9% in esophageal and gastric resections [7,17]. However, clinical data for HPB surgery is rather lacking [7]. Following the encouraging results in colorectal resections, we initiated the preoperative SDD administration in our institution in patients scheduled for pancreatic surgery in January 2019 and evaluated our results after 18 months of use.

The implementation of SDD in surgery was initially triggered by the knowledge of the gut microbiome and its impact on infectious conditions [1]. The gut microbiome in humans is an intestinal microbial ecosystem consisting of bacteria, viruses, and fungi defined by a stable balance of pathogenic and non-pathogenic microorganisms in healthy individuals, called eubiosis [1,18,19]. Recently, a number of studies suggested that an abundance of pathological bacteria and damage of the gut environment balance (a condition known as dysbiosis) may lead to postoperative complications [20]. Furthermore, it was shown that surgery on the gastrointestinal tract itself causes changes in the proliferation of the pathogenic species, which triggers infectious conditions by damaging the mucosal integrity of the gastrointestinal tract. Some pathogens, including Pseudomonas aeruginosa, Enterococcus faecalis, and Serratia marcescens, are increasingly present when anastomotic insufficiencies occur [21].

Langheinrich et al. focused on the role of the gut microbiome as a contributive factor for postoperative complications in pancreatic surgery [22]. They observed a higher abundance of Gram-positive bacteria, particularly Enterococcus spp., in bile, pancreatic, and duodenum tissue when POPF occurred. A Japanese study group identified bacteria such as Staphylococcus epidermidis, Enterococcus faecalis, Escherichia coli, Klebsiella pneumonie, and others in pancreatic juice in cases of acute pancreatitis and tumor lesions [23]. Further research by Rogers et al. addressed the potentially contributing effect of Klebsiella pneumonie on POPF [24]. These data postulate the association between short-term complications in pancreatic resections and the gut microbiome. In our analysis on the bacterial spectrum in the bile cultures, a major shift to fewer Gram-negative bacteria and Candida spp. in the SDD group was found. SDD is based on oral antibiotics and antimycotics, eliminating potentially pathogenic bacteria and fungi in the intestinal tract. Hence, even short-term perioperative oral antibiotic administration may lead to less bile contamination. The latter, meanwhile, was found to be an important predictor of postoperative morbidity in several studies [25,26,27]. Thus, SDD application preoperatively might improve postoperative outcomes.

Bile duct stenting prior to pancreatic resections is still a matter of debate with controversial results in clinical research, considering complication rates and wound infections [7,25,28,29]. Since alloplastic material is often covered with bacterial biofilm, it is known to increase the rate of complications due to contamination of the bile and subsequent infectious conditions [7,26,27,30]. Data are very scarce and controversial concerning the association of bacterial contamination of the bile with the development of POPF [24,30]. Although no strong statistical causality has been demonstrated so far, the virulence of some pathogens is being discussed as a possible contributing factor to POPF [24,27,30]. Our data demonstrate a global shift towards a lower bacterial bile load in stent patients, with a significant reduction of Gram-negative Enterobacter and Citrobacter spp. after the administration of SDD. However, a subgroup analysis driven by the duration of stenting prior to surgery was not accomplished due to the small number of patients.

Enterobacter has already been reported in some studies to be associated with the development of postoperative complications [1]. Consequently, the reduction of Gram-negative pathogenic bacteria may also reduce the incidence of postoperative complications in pancreatic surgery.

In our study on a propensity score-matched patient cohort, global results showed no statistical difference for postoperative complications in either group, with and without decontamination. Major complications defined as CDC >2 and, especially, deep organ abscesses showed a slightly decreasing tendency in the SDD group. This is in line with data for upper GI surgery, although no significant benefit on mortality and SSIs was seen after the administration of SDD [15,16]. In the literature there is a strong assumption that bacterial load is an important risk factor for the development of pancreatic fistula [19,22]. We did not perform a subgroup analysis and therefore cannot provide a conclusion about the association between abscesses and POPF.

Notably, LOS and length of ICU stay were significantly reduced after the preoperative introduction of SDD administration. A possible contributing aspect is the adoption of the enhanced-recovery-after-surgery (ERAS) concept in our institution, containing less opioid analgesia, modified drain management, and diet changes. However, a stepwise statistical comparison among all enclosed years revealed no statistically significant changes in LOS and ICU stay without regarding SDD administration. Furthermore 30-day and 90-day mortality were slightly increased in the SDD group compared to the non-SDD group. This is in contrast to the overall complication rate, as mentioned above. Therefore, the exact effect explaining this change remains unclear. Ultimately, an unknown bias must be assumed in a retrospective study.

Overall, an impact of the gut microbiome on postoperative complications in pancreatic surgery is emerging. The use of SDD in colorectal surgery is evident; however, currently, there is not enough data regarding its relevance in HPB surgery.

Our study aimed to analyze the first results after implementing SDD for pancreatic resections with a propensity score-matched cohort. However, some possible bias must be considered when interpreting the results. First, perioperative antibiotic prophylaxis was progressively switched to piperacillin/tazobactam during the first year (2019) of SDD application to address suspected resistance [31]. Second, we started in 2021 with a prospective observational study referring postoperative contaminated intraabdominal fluid collections, wherein patients after pancreatoduodenectomy were irrigated with 5 l saline solution before wound closure.

The main limitation of the study is its retrospective character. The available data are shown transparently in the tables in accordance with the STROBE statement. There is an increased risk for recall bias, especially concerning patient data and operation variables. As we present data from a single center, a generalization of our results is limited. Despite the small patient number, our results demonstrate signs of benefits for patients with pancreatic resections compared to a propensity score-matched cohort. In addition, this is the first study investigating the impact of SDD after pancreatic resection in a propensity score-matched fashion. In order to address substantial short-term effects, a bigger patient cohort and prospective data is urgently needed.

## 5. Conclusions

The use of SDD leads to globally lower bacterial and mycotic loads in bile duct cultures for patients undergoing pancreatic head resections. This is even more evident in patients with a preoperative biliary drainage. A shift towards fewer major postoperative complications and deep organ abscesses and less fluid collection is visible. However, more data is needed to investigate the profound impact of patients’ short- and long-term outcome after pancreatic surgery.

## Figures and Tables

**Table 1:** Basic patient characteristics.

	Overall	Non-SDD	SDD	*p* Value
patients [*n* (%)]	200	100 (50)	100 (50)	
sex [*n* (%)]				
m	122 (61)	61 (61)	61 (61)	1
w	78 (39)	39 (39)	39 (39)	
age [years] (IQR)	65.3 (56.9–75)	65.3 (56.9–73.6)	66 (56.3–76)	0.91
median BMI [kg/m²] (IQR)	25 (22.6–28)	25.2 (23.6–28.4)	24.7 (22.2–27.4)	0.07
smoking [*n* (%)]	59 (29.5)	27 (27)	32 (32)	0.44
ASA score [*n* (%)]				
1	6 (3)	3 (3)	3 (3)	0.24
2	77 (38.5)	44 (44)	33 (33)	
3	114 (57)	52 (52)	62 (62)	
4	2 (1)	0	2 (2)	
diabetes [*n* (%)]	70 (35)	36 (36)	34 (34)	0.76
insulin-dependent diabetes (IDDM) [*n* (%)]	46 (23)	22 (22)	24 (24)	0.74
preoperative biliary drainage [*n* (%)]	81 (40.5)	38 (38)	43 (43)	0.47
histopathological analysis [*n* (%)]				
malignancy	142 (71)	68 (68)	74 (74)	0.08
chronic pancreatitis	38 (19)	17 (17)	21 (21)	
cystic pancreatic lesion	9 (4.5)	7 (7)	2 (2)	
benign tumor	3 (1.5)	1 (1)	2 (2)	
operations performed [*n* (%)]				
pylorus-preserving PD (PPPD)	143 (71.5)	75 (75)	68 (68)	0.18
classic PD (cPD)	38 (19)	14 (14)	24 (24)	
duodenum-preserving pancreatic head resection (DPPHR)	19 (9.5)	11 (11)	8 (8)	
intraoperative portal vein resected [*n* (%)]	64 (32)	26 (26)	38 (38)	0.07
Intraoperative arterial resection [*n* (%)]	18 (9)	6 (6)	12 (12)	0.14
fistula risk score (FRS) [IQR]	3 (2–5)	3 (2–5)	3 (2–5)	0.96

**Table 2:** Intraoperative bile duct smear test.

	Overall	Non-SDD	SDD	*p* Value
patients [*n* (%)]	200	100 (50)	100 (50)	
intraoperative smear test [*n* (%)]				
available	137 (68.5)	50 (50)	87 (87)	**<0.001**
positive	85 (62)	34 (68)	51 (58.6)	0.28
positive (patients without bile duct stent)	21 (29.6)	11 (44)	10 (21.7)	0.05
absolute number of bacteria in bile duct culture [*n*] (IQR)	2 (0–3)	2 (0–4)	1 (0–3)	**0.04**
bacterial load [*n* (%)]				
bile duct cultural bacterial load “none”	52 (38)	16 (32)	36 (41.4)	0.37
bile duct cultural bacterial load “occasional”	9 (6.6)	4 (8)	5 (5.7)	
bile duct cultural bacterial load “numerous”	16 (11.7)	4 (8)	12 (13.8)	
bile duct cultural bacterial load “massive”	60 (43.8)	26 (52)	34 (39.1)	

**Table 3:** Outcome analysis.

	Overall	Non-SDD	SDD	*p*-Value
patients [*n* (%)]	200	100 (50)	100 (50)	
length of hospital stay (LOS) (IQR)	16 (12–27)	19 (14–30)	14 (10–22.8)	**<0.001**
length of intensive care unit stay (ICU stay) (IQR)	2 (1–5)	4 (2–6.5)	2 (1–3)	**<0.001**
ICU readmission [*n* (%)]	32 (16)	16 (16)	17 (17)	0.85
postoperative complication [*n* (%)]				
CDC > 2 [*n* (%)]	95 (47.5)	49 (49)	46 (46)	0.67
in-hospital mortality [*n* (%)]	16 (8)	8 (8)	8 (8)	1
30-day mortality [*n* (%)]	24 (12)	8 (8)	16 (16)	0.08
90-day mortality [*n* (%)]	36 (18)	14 (14)	22 (22)	0.14
surgical site infection (SSI) [*n* (%)]	44 (22)	20 (20)	24 (24)	0.5
deep organ space abscesses / fluid collection [*n* (%)]	63 (31.5)	36 (36)	27 (27)	0.17
clinically relevant pancreatic fistula (POPF) [*n* (%)]	40 (20)	17 (17)	23 (23)	0.31
postpancreatectomy hemorrhage (PPH) [*n* (%)]	31 (15.5)	13 (13)	18 (18)	0.34
delayed gastric emptying (DGE) [*n* (%)]	46 (23)	22 (22)	24 (24)	0.74
postoperative positive blood cultures [*n* (%)]	25 (12.5)	13 (13)	12 (12)	0.83

## Data Availability

The data are available from the corresponding author upon request.

## Abbreviations

**SDD**—selective decontamination of the digestive tract; **HPB**—hepato-pancreato- biliary; **POPF**—postoperative pancreatic fistula; **SSI**—surgical site infection; **MBP**—mechanical bowel preparation; **STROBE**—STrengthening the Reporting of OBservational studies in Epidemiology; **DPPHR**—duodenum-preserving pancreatic head resection; **PPPD**—pylorus-preserving pancreatic head resection; **cPD**—classic pancreaticoduodenectomy; **BMI**—body mass index; **ASA**—American Society of Anesthesiologists; **CDC**—Clavien–Dindo classification; **DGE**—delayed gastric emptying; **PPH**—postpancreatectomy hemorrhage; **LOS**—length of hospital stay; **ICU**—intensive care unit; **IQR**—interquartile range; **AI**—anastomotic insufficiency.
